# ggmotif: An R Package for the extraction and visualization of motifs from MEME software

**DOI:** 10.1371/journal.pone.0276979

**Published:** 2022-11-03

**Authors:** Xiang Li, Linna Ma, Xinyue Mei, Yixiang Liu, Huichuan Huang

**Affiliations:** 1 State Key Laboratory for Conservation and Utilization of Bio-Resources in Yunnan, Yunnan Agricultural University, Kunming, China; 2 Key Laboratory for Agro-Biodiversity and Pest Control of Ministry of Education, Yunnan Agricultural University, Kunming, China; University of Cyprus, CYPRUS

## Abstract

MEME (Multiple Em for Motif Elicitation) is the most commonly used tool to identify motifs within deoxyribonucleic acid (DNA) or protein sequences. However, the results generated by the MEMEare saved using file formats .*xml* and .*txt*, which are difficult to read, visualize, or integrate with other widely used phylogenetic tree packages, such as *ggtree*. To overcome this problem, we developed the *ggmotif* R package, which provides two easy-to-use functions that can facilitate the extraction and visualization of motifs from the results files generated by the MEME. *ggmotif* can extract the information of the location of motif(s) on the corresponding sequence(s) from the .*xml* format file and visualize it. Additionally, the data extracted by *ggmotif* can be easily integrated with the phylogenetic data. On the other hand, *ggmotif* can obtain the sequence of each motif from the .*txt* format file and draw the sequence logo with the function *ggseqlogo* from the *ggseqlogo* R package. The *ggmotif* R package is freely available (including examples and vignettes) from GitHub at https://github.com/lixiang117423/ggmotif or from CRAN at https://CRAN.R-project.org/package=ggmotif.

## Introduction

Motifs are regions (or subsequences) of deoxyribonucleic acid (DNA) or protein sequences with a specific structure. These motifs generally contain functionally important sequences and are therefore often used as a base to classify proteins.

Two main approaches can be used to search and discover motifs: the enumerative approach and the probabilistic approach [1]. The MEME utilizes a probabilistic approach and is one of the most widely used software programs for the identification of novel “sequences” in sets of biological sequences [2–6]. The results generated by MEME command line software include figures of each motif and three other files, an .*xml* file, a .*txt* file, and an .*html* file. The .*html* file is a nice output for visual inspection of the results. However, the user cannot freely combine the figures they want from the .*html* file and directly generate them with MEME. The .*txt* file and .*xml* file contain almost all the information of the .*html* file. However, these files are difficult to read and cannot be used to directly generate figures and tables that meet the quality demands of publication standards. Some R/Bioconductor packages have been developed to process the results from MEME [7–9]. However, these R/Bioconductor R packages cannot parse the position of each motif on the corresponding sequences from the latest MEME. For example, the functions read_meme and importMeme from the Bioconductor packages universalmotif and memes, respectively, can only parse the information from the .txt file and cannot extract the position information. Their result is a list that is slightly unfriendly for the user. Additionally, the results produced by the MEME are difficult to combine with other downstream analysis software, such as *ggtree* [10]. The Bioconductor package motifstack can plot stacks with a hierarchical tree of the corresponding motifs [9]. However, in many cases, the user wants to visualize the phylogenetic tree with the corresponding position of each motif on the corresponding sequences.

Therefore, in this study, we developed the easy-to-use R package *ggmotif* to facilitate the extraction and visualization of results from the multiple file formats generated by the MEME and facilitate the integration of the data with other phylogenetic visualization tree packages, such as *ggtree*.

## Materials and methods

In this study, we picked up the sequences of the AP2 gene family as example. The AP2 gene family, a large gene family of transcription factors, belongs to the AP2/ERF superfamily that plays important role during the lifespan of plant [11]. The sequences of the AP2 gene family of *Arabidopsis thaliana* were downloaded from PlantTFDB [12]. MEME (V5.4.1) was used to identify motifs with the parameters -protein -o meme_out -mod zoops -nmotifs 10 -minw 4 -maxw 7 -objfun classic -markov_order 0. Clustalo (V1.2.4) [13] and FastTree (V2.1.10) [14] were used to align the sequences and construct the phylogenetic tree. The bash code can be found at GitHub (https://github.com/lixiang117423/ggmotif).

**Fig 1:**
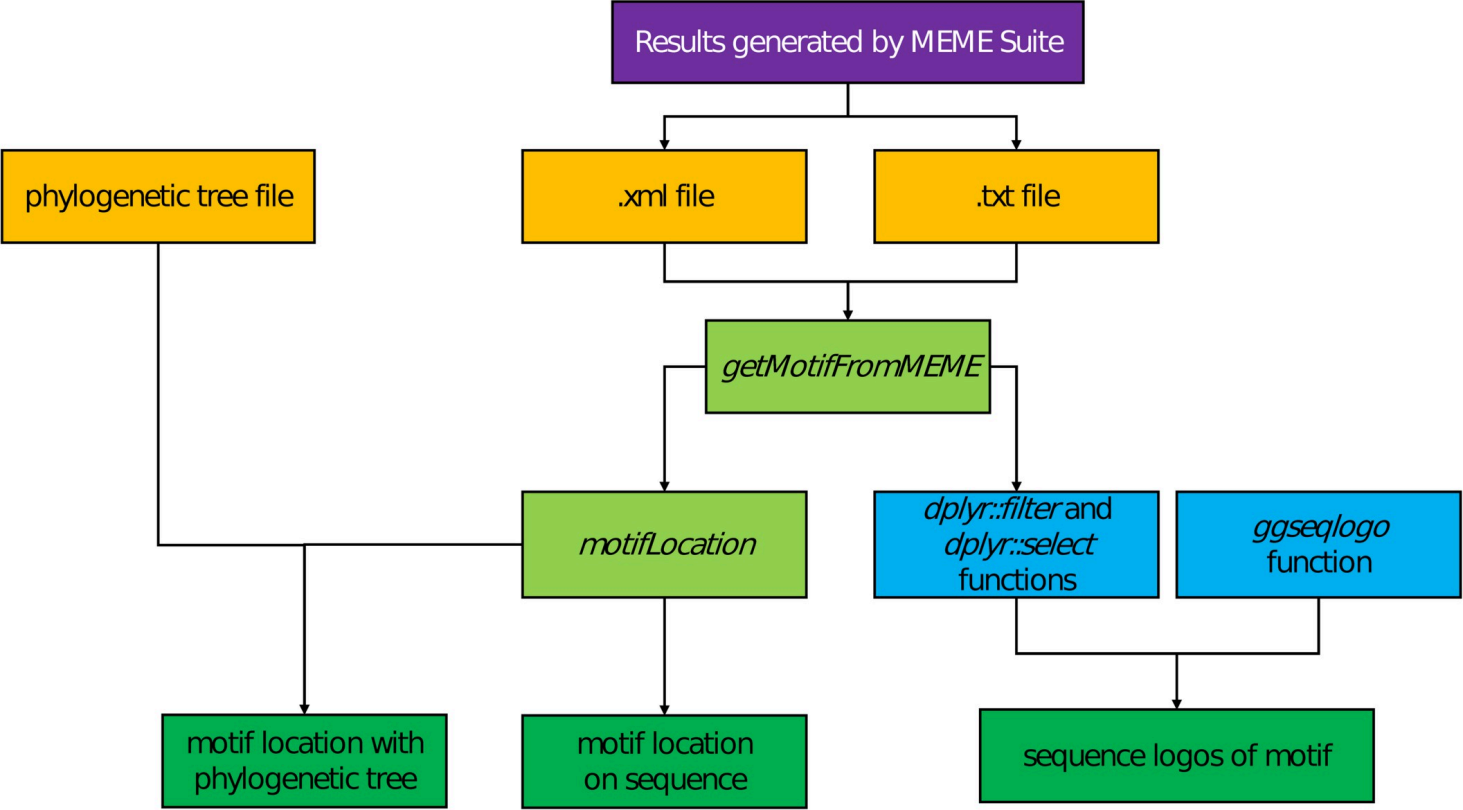
*ggmotif* workflow. The *ggmotif* workflow consists of several functions, including the *ggmotif* package, *ggtree* package, and *ggseqlogo* package.

The *ggmotif* workflow is shown in Fig 1. Briefly, the MEME can generate several files, including an .*xml* file and a .*txt* file containing the information of the location on sequence (s) and the sequence of motifs, respectively. The function *getMotifFromMEME* can extract the information described above and convert it to dataframe-type data objects that are user-friendly to biologists in the R environment. After that, the function *motifLocation* can be used to visualize the location of motif(s) on sequence(s), such as gene(s). If the user wants to plot the location of motif(s) on sequence(s) with the corresponding phylogenetic tree, the parameter *tree* will be used to meet the need with the help of *ggtree*. The MEME can make two type files, an .*eps* format figure and a .*png* format figure for motif sequence logo. That is enough for some users but not for others. After extracting the information of sequences of motifs, the user can filter or select motif(s) of interest to visualize with the function *ggseqlogo* from the *ggseqlogo* package [15].

## Results

### Obtaining motif information from files generated by MEME

The results files generated by the MEME were stored using the .*xml* file and .*txt* file formats. The .*xml* format file contains the motifs’ information on the input sequence(s), and the .*txt* format file is used to store the sequences of all identified motif(s). The function *getMotifFromMEME* can be used to extract motif information from the .*xml* and .*txt* files by simply inputting the .*xml* format file or .*txt* format file to obtain the dataframe containing the corresponding information (S1 and S2 Tables). The result extracted from the .*xml* format file included a set of information, such as the id and length of input sequence(s), content for the motif, start position, end position, and p value, and so on. The user can filter motif(s) by some parameter, for instance, motif id, p value, e-value, and/or Bayes threshold. The other information from the .*txt* format file mainly included motif id and sequence. Users can filter or select motif(s) of interest for visualization. The functions *importMeme* from the Bioconductor package memes can parse information from the .*txt* file. However, the extracted table didn’t contain the location of motifs on corresponding sequences (S3 Table). The widely used Bioconductor packages memes or universalmotif both cannot handle the .*xml* file.

### Visualization of motif location on sequences

**Fig 2:**
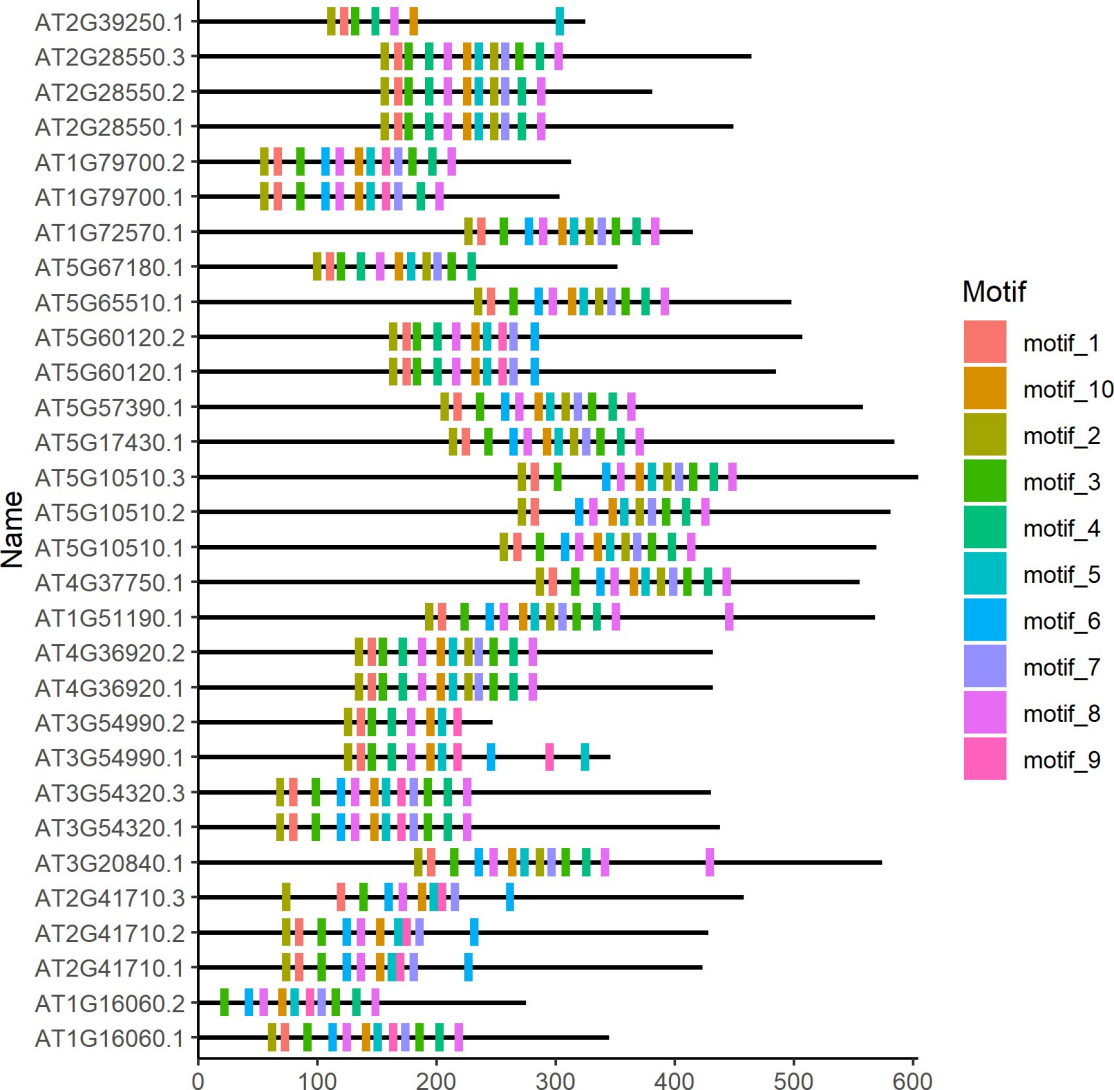
The location of motifs on the corresponding sequences.

After obtaining the motif information, the user can then visualize the sequence logos or the location of the motifs on the corresponding sequences. Conversely, the function *motifLocation* can be used to visualize the location of the motif(s) on the corresponding sequence(s) (Fig 2). The reference code is shown below:

library(ggmotif)
filepath <- system.file("examples", "meme.xml", package = "ggmotif")
motif_extract <- getMotifFromMEME(data = filepath, format = "xml")
motif_plot <- motifLocation(data = motif_extract)
motif_plot

### Visualization of motif location on sequences with a phylogenetic tree

**Fig 3:**
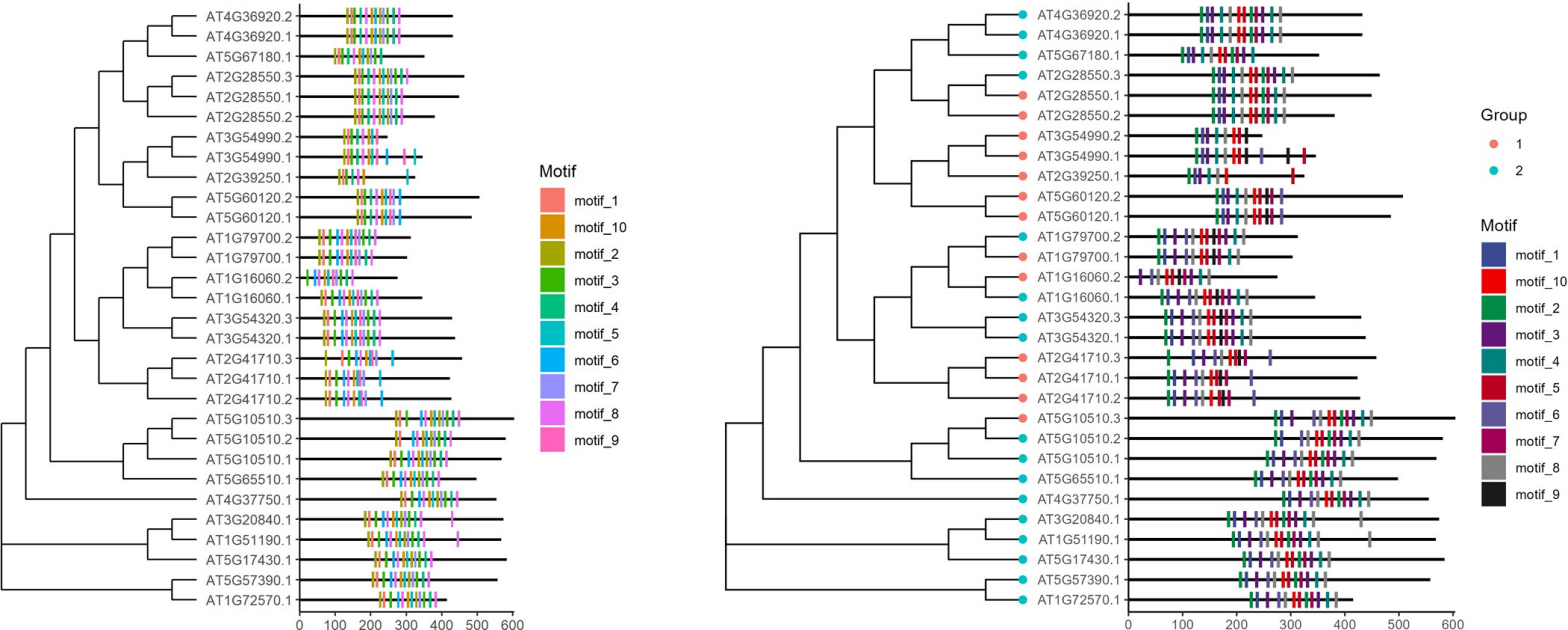
The location of motifs on the corresponding sequences (right panel) and the corresponding phylogenetic tree (left panel). The light blue and orange dots in Fig 3B represent the number of AP2/ERF domains in the corresponding sequences.

If the user has the corresponding phylogenetic tree, the id(s) is the same as the id(s) in the sequences used to identify motifs using MEME, the function *motifLocation* with parameter *tree* can be used to visualize the location of motif(s) on the corresponding sequence(s) after sorting the sequence(s) order with a phylogenetic tree. For example, as shown in Fig 3A, AT4G36920.2 is the id of the sequence used to identify motifs using MEME and construct the phylogenetic tree. So do other sequences. The figure generated by the parameter *tree* is the same as the above, but the order of sequences(s) is adjusted to fit with the phylogenetic tree (Fig 3A). The id of the phylogenetic tree requires that all formats supported by Bioconductor package *ggtree* must be consistent with the sequences inputted to MEME. If other information of phylogenetic tree is available, the parameter *tree*.*anno* can be used to plot the tip-point of phylogenetic tree (Fig 3B). As shown in demo result (Fig 3B), when the motifs positions are similar, the genetic relationship between the sequences is also similar. The reference code is shown below:

library(ggmotif)
filepath <- system.file("examples", "meme.xml", package = "ggmotif")
treepath <- system.file("examples", "ara.nwk", package = "ggmotif")
motif_extract <- getMotifFromMEME(data = filepath, format = "xml")
motif_plot <- motifLocation(data = motif_extract, tree = treepath)
motif_plot
tree.anno.path <- system.file("examples", "tree.anno.txt", package = "ggmotif")
tree.anno = data.table::fread(tree.anno.path) %>%
 dplyr::mutate(Group = as.character(Group))
motif_plot <- motifLocation(data = motif_extract, tree = treepath, tree.anno = tree.anno)
motif_plot +
 ggsci::scale_fill_aaas()

## Conclusion

The search and characterization of motifs for the given sequence(s) provides valuable insights into their role in biological regulation. Unlike previously developed tools, such as TBtools [16], *ggmotif* gives the user familiar with command line tool R a chance to extract and visualize motif information on the corresponding sequence(s). The Bioconductor package motifStack can plot motifs using tree-like structures, but it cannot visualize motif locations on corresponding sequences with phylogenetic trees. In other tools, it is difficult for the user to filter or select the motif of interest to visualize, and the user must almost handle the whole process if the motif of interest is changed. Overall, *ggmotif* is a user-friendly R package that provides functions to extract and visualize motif information from MEME.

## Supporting information

S1 Table The extracted information from the .*xml* format file by the function *getMotifFromMEME*. (XLSX)

S2 Table The extracted information from the .*txt* format file by the function *getMotifFromMEME*. (XLSX)

S3 Table The extracted information from the .txt format file by the funxtion *importMeme* from R package memes with parameters “combined_sites = TRUE”. (XLSX)

S1 Fig Sequence logs of a single motif. (TIFF)

S2 Fig Sequence logs of all motifs. (TIFF)
