# Supplementary figures and images for: ggmotif: An R Package for the extraction and visualization of motifs from MEME software
Source: PLoS One. 2022 Nov 3;17(11):e0276979. doi: 10.1371/journal.pone.0276979 (PMC9632824; doi:10.1371/journal.pone.0276979)

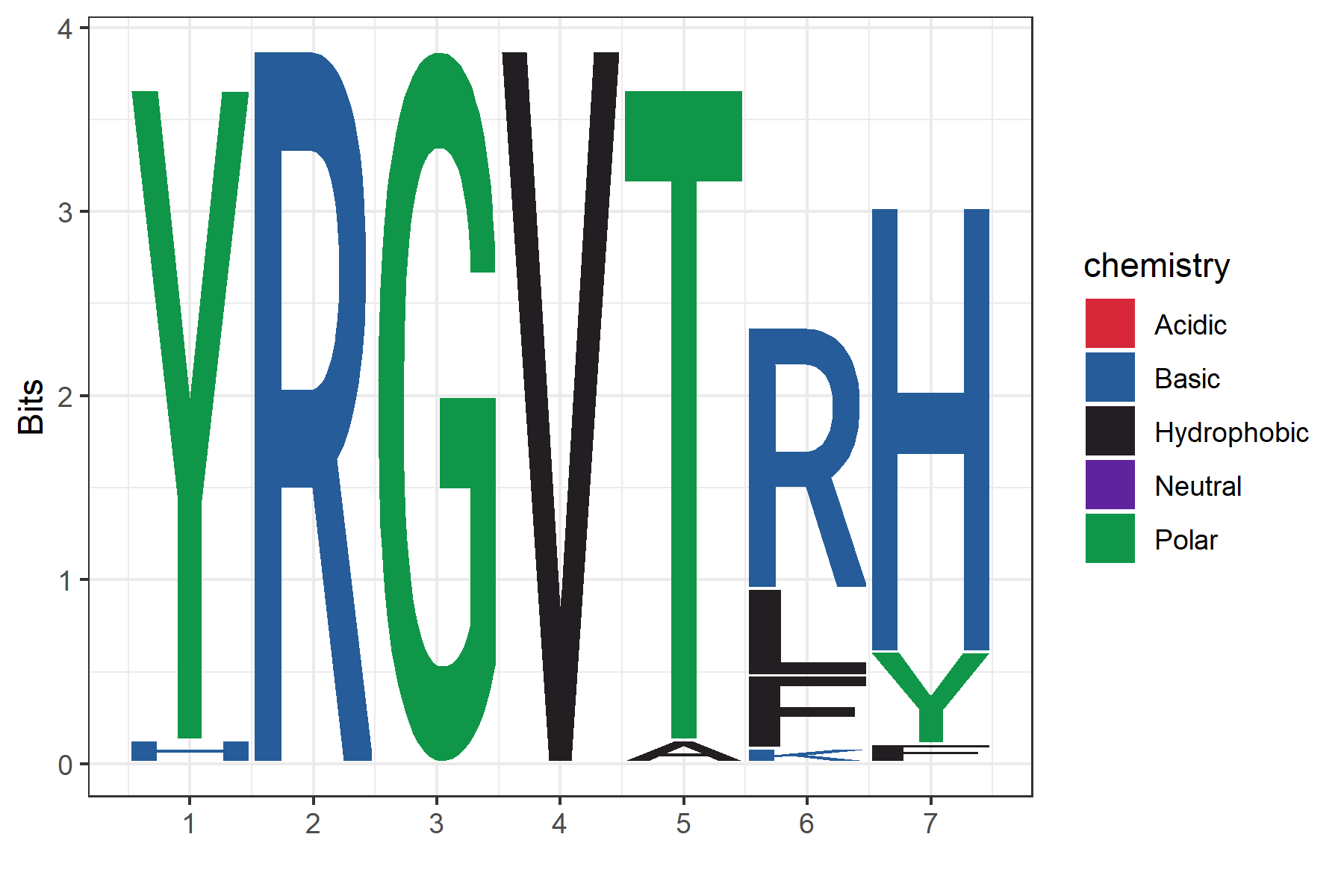

Supplement: S1 Fig — (TIFF) [file pone.0276979.s004.tiff]
